# Self-reported diagnosis of heart disease: results from the SHIELD study

**DOI:** 10.1111/j.1742-1241.2009.02049.x

**Published:** 2009-05

**Authors:** S J Lewis, K M Fox, S Grandy, for the SHIELD Study Group

**Affiliations:** 1 Northwest Cardiovascular InstitutePortland, OR, USA; 2 Strategic Healthcare SolutionsLLC, Monkton, MD, USA; 3 AstraZeneca LPWilmington, DE, USA

## Abstract

**Objective:**

This study evaluated the self-reported method of diagnosis of heart disease (HD) to elucidate whether diagnosis is occurring at early, presymptomatic stages as recommended by the prevention guidelines.

**Methods:**

Respondents to the 2006 survey in the US population-based Study to Help Improve Early evaluation and management of risk factors Leading to Diabetes (SHIELD) reported whether a physician told them that they had HD, including heart attack, angina, heart failure, angioplasty or heart bypass surgery. Self-report of age at diagnosis, specialty of physician who made the diagnosis and whether the diagnosis was made after having symptoms, during routine screening or while being treated for another health problem were assessed. Year of diagnosis was categorised into 3-year intervals from 1985 to 2006. Individuals with HD diagnosis with and without type 2 diabetes mellitus (T2DM) were compared using chi-square tests.

**Results:**

Of 1573 respondents reporting a diagnosis of HD, > 87% were white, > 49% were men and 38% had T2DM. Approximately 19% of respondents reported that their HD diagnosis was made during routine screening. A significantly greater percentage of HD respondents with T2DM reported the diagnosis being made based on symptoms (54%) and while being treated for another health problem (22%) compared with respondents without diabetes (48% symptoms and 15% other health problem, p > 0.05). HD was diagnosed primarily by cardiologists (> 60%) and family doctors (> 25%).

**Conclusion:**

There remains a missed opportunity to diagnose HD at earlier stages through routine screening or during treatment of other health conditions such as diabetes, as many individuals were not diagnosed until they were symptomatic.

**What’s known**

Coronary heart disease is a leading cause of death and disability worldwide. Recent evidence-based scientific guidelines have recommended increased screening for heart disease and risk factors.

**What’s new**

This article provides evidence from a large sample of individuals in the community that there remains a missed opportunity to diagnose heart disease through routine screening or during treatment of other health conditions. More than 50% of individuals reported that their heart disease was diagnosed after symptoms arose.

## Introduction

Coronary heart disease (CHD) is the leading cause of death in the United States, accounting for more than 450,000 deaths in 2004 (1), largely because of myocardial infarction and sudden cardiac death (2). Approximately 15.8 million Americans aged 20 years and older have CHD (1), although many individuals are asymptomatic and go undiagnosed until the disease is in an advanced state, often after experiencing a myocardial infarction (2). The tremendous burden of CHD has led to the development of guidelines and policies on prevention in the USA, encompassing a larger effort to prevent and treat cardiovascular disease (CVD), including stroke (3–6).

Because of the often asymptomatic nature of CHD and CVD in general, assessing CVD risk factors is usually the starting point for determining a patient’s actual risk for CHD or CVD (7,8). Major risk factors include tobacco smoking, high blood cholesterol, high blood pressure, diabetes, obesity and overweight, physical inactivity and increasing age (2,9). Comprehensive risk factor screening and follow-up by a primary care provider (PCP) or other physician are generally recommended every 2–5 years for every adult, beginning at the age of 20 years (4,5,10,11). More specific screening recommendations exist for those at increased risk, such as those with type 2 diabetes mellitus (T2DM), who have twice the risk of having a myocardial infarction or stroke than the general public (4).

Although the burden of CHD is clear, screening of risk factors and awareness of CHD and CVD in US are less than optimal. National efforts have been under way to promote CVD and CHD risk factor screening, with a specific effort to achieve cholesterol screening in 80% of American adults (12). According to the Centers for Disease Control, the percentage of those screened for high blood cholesterol in the USA increased from 67.6% in 1991 to 73.1% in 2003 (12). Even so, this suggests that increased awareness and screening efforts for CVD, and CHD specifically, are still needed. A study by Mosca et al. (13) demonstrated that awareness of heart disease (HD) among women has increased, although only half of women are aware that HD is their leading cause of death. Physician awareness and adherence to CVD and CHD guidelines also vary. In a study by Mosca et al. (14), obstetricians and gynaecologists, most of whom provide primary care to their patients, were substantially less aware of national cholesterol and blood pressure management guidelines than PCPs or cardiologists. Physicians were also more likely to assign lower CVD risk categories to women who had similar calculated risks to men (14). Thus, there appears to be multiple factors that contribute to CHD being diagnosed at later stages, when symptoms (e.g. angina) occur.

This study was designed to determine if the self-reported method of diagnosis of HD has changed in recent years (since 2001) as several guidelines have been published highlighting the need for primary and secondary prevention. We hypothesised that routine screening for HD would be greater in the intervals of 2001 and later compared with 2000 and before, because the AHA/ACC primary prevention guidelines (5,6) and the National Cholesterol Education Program (NCEP) Adult Treatment Panel (ATP) III (15) were published in 2001–2002 and the AHA guidelines for CVD prevention in women were published in 2004 (10). The study findings should provide insight into whether HD is being detected through routine screening, including risk factor screening, or whether individuals are continuing to be diagnosed at later, symptomatic stages.

## Methods

A cross-sectional analysis of survey data from the 2006 Study to Help Improve Early evaluation and management of risk factors Leading to Diabetes (SHIELD) survey was conducted to determine the method of diagnosis of HD.

### SHIELD surveys

SHIELD has three phases extending over 5 years: (i) an initial screening phase to identify cases of interest in the general population; (ii) the baseline survey to follow-up identified individuals with a questionnaire about health status, health knowledge and attitudes, and current behaviours and treatments; and (iii) four additional annual surveys to follow disease progression in those with established diabetes as well as the rate of transition from at risk to a diagnosis of diabetes. The SHIELD survey methodology has been described in detail previously (16,17).

The screening survey was mailed on 1 April 2004 to a stratified random sample of 200,000 US households, representative of the US population for geographic residence, household size and income, and age of head of household, identified by the Taylor Nelson Sofres National Family Opinion (TNS NFO) panel. The screening survey consisted of 12 questions designed to identify individuals with diabetes mellitus and those with cardiometabolic risk factors. The head of household completed the screening questionnaire for up to four adult (aged ≥ 18 years) household members. A response rate of 63.7% was obtained from 127,420 households (containing 211,097 adults).

The baseline survey was mailed in September and October 2004 to a representative sample of individuals, independently sampled (*n* = 22,001), who were identified in the screening survey as having type 1 diabetes mellitus, T2DM or one of five cardiometabolic risk factors [abdominal obesity, body mass index (BMI) ≥ 28 kg/m^2^, diagnosis of dyslipidaemia, diagnosis of hypertension, or history of CVD, including HD/heart attack, narrow or blocked arteries, stroke, heart bypass surgery, angioplasty or surgery to clear arteries]. Each respondent group was balanced to be representative of that population for age, gender, geographic region, household size and income as the US population, based on the weighted screening data; a random sample from each group was then selected and sent the baseline survey. A response rate of 71.8% was obtained (*n* = 15,794).

### Follow-up surveys

**Figure 1:**
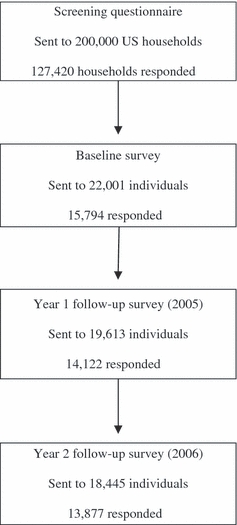
Flow of SHIELD surveys

In August 2005, the first annual follow-up survey was mailed to all individuals selected for the baseline survey who were still enrolled in the TNS NFO panel (*n* = 19,613). The second annual follow-up survey was mailed in July 2006 to individuals who had returned either or both the baseline and first annual questionnaires (*n* = 18,445). A 75% response rate was obtained for the 2006 follow-up survey (*n* = 13,877). Figure 1 shows the progression of the SHIELD surveys over time.

### Risk factors

Five cardiometabolic risk factors were identified through epidemiological studies and expert opinion (15,18) to be associated with CHD. SHIELD respondents reported their height and weight and whether they had ever been told by a doctor that they had cholesterol problems of any type, high blood pressure/hypertension, or history of CVD (defined as HD/heart attack, narrow or blocked arteries, stroke, coronary artery bypass graft surgery, angioplasty/stents to clear arteries). Respondents were provided with a measuring tape and while standing were asked to hold the tape measure loosely around their waist at the level of their navel (belly button) to determine waist circumference. This information was used to define the five risk factors as: (i) abdominal obesity (waist circumference: men, ≥ 97 cm; women, ≥ 89 cm), (ii) BMI ≥ 28 kg/m^2^, (iii) reported diagnosis of dyslipidaemia, (iv) reported diagnosis of hypertension, and (v) history of CVD. Other risk factors for CHD and CVD were examined among the respondents and included smoking (current, past and never smoked), obesity (classified underweight/normal weight as BMI ≤ 24.9 kg/m^2^, overweight as BMI 25.0–29.9 kg/m^2^, obesity as BMI ≥ 30 kg/m^2^) and physical activity [highly active, minimally active and inactive based on the International Physical Activity Questionnaire (IPAQ) (19)].

### HD diagnosis

Respondents who reported HD/heart attack, including angina, heart failure, angioplasty and/or heart bypass surgery were identified as having HD. Individuals with HD were asked to indicate the age at which they were diagnosed with HD. Subtracting age at HD diagnosis from the respondent’s current age provided estimates for the timing (year) of the HD diagnosis. Year of HD diagnosis was categorised into 3-year intervals to capture changes before and after the guidelines on CHD screening and prevention (3,5,6,10,15,20). This resulted in the following eight HD diagnosis intervals: 2004 or later, 2001–2003, 1998–2000, 1995–1997, 1992–1994, 1989–1991, 1986–1988, or 1985 or earlier. As noted previously, because the AHA/ACC primary prevention guidelines (5,6) and the NCEP ATP III (15) were published in 2001–2002 and the AHA guidelines for CVD prevention in women were published in 2004 (10), it was hypothesised that screening for HD would be greater in the intervals of 2001 and later compared with 2000 and before.

Respondents who self-reported a diagnosis of HD were also asked ‘how did you find out that you had HD.’ Response options included ‘during routine screening/lab work (blood test, etc.) ordered by my doctor’ (i.e. routine screening); ‘when I was tested for it after having some health symptoms’ (i.e. symptoms); or ‘when I was being treated for another health problem’ (i.e. other health problems). Respondents were permitted to select multiple answers. With these methods of diagnosis, it was determined whether CHD and CVD screening and prevention recommendations have led to a trend toward increased diagnoses for HD as a result of routine screening or whether the trend continues to reflect patients being diagnosed after experiencing symptoms of HD or having a major CVD event. Respondents were not asked about specific screening or blood tests such as lipid levels, blood pressure or ECG or cardiac stress tests.

Individuals were also asked to indicate the specialty of the physician who made their diagnosis (e.g. family doctor/general practitioner, cardiologist or other specified physician), as it would be important to observe whether the promotion of guidelines has resulted in increased screening and diagnosis by certain specialists other than cardiologists.

### Statistical analyses

Respondents with HD were stratified into individuals with and without T2DM because T2DM confers higher risk for CHD and is considered a CHD risk equivalent condition by NCEP ATP III (15). Comparisons between HD respondents with and without T2DM were made to determine if the diagnosis of HD was made more frequently through screening or while being treated for another health problem (i.e. T2DM) for respondents with T2DM. Descriptive statistics for sociodemographic characteristics and diagnosis information (age, year of diagnosis, method and physician specialty) were provided. Comparisons between respondents with HD and no T2DM vs. HD with T2DM were made using chi-square tests for proportions and *t*-tests for comparison of means. A p < 0.05 was considered statistically significant.

## Results

There were 1573 (of 13,877) respondents from the 2006 SHIELD survey who reported a diagnosis of HD and provided their age at time of diagnosis. Approximately 62% (*n* = 973) of these HD respondents did not have diabetes mellitus (i.e. type 1, type 2 or gestational diabetes), while 38% (*n* = 600) of these respondents had HD and T2DM.

### Demographics

In the non-diabetes group, HD respondents were predominantly male (59.0%), white (91.3%) and of non-Spanish heritage (99.0%) (Table 1). The respondents with HD and T2DM were significantly younger (p = 0.0006) and fewer were men (p = 0.0002) compared with the HD respondents without diabetes (Table 1). The two groups were similar in race and annual household income (p > 0.05).

**Table 1:** Sociodemographic characteristics of SHIELD respondents diagnosed with HD (*n*= 1573)

Characteristics	HD without diabetes (*n* = 973)	HD + type 2 diabetes mellitus (*n* = 600)
Gender, men, %	59.0**	49.3
Age, years, mean (SD)	69.0 (11.5)**	67.0 (11.1)
**Race, %†**		
White	91.3	87.8
Black	4.8	7.3
Other, including Asian/PacificIslander, American Indian,Eskimo, others	1.1	1.5
Spanish/Hispanic heritage, %	1.0	1.8
**Annual household income, %**		
< $20,000	25.4	28.5
$20,000-$34,999	21.7	20.8
$35,000-$54,999	19.6	23.3
$55,000-$84,999	16.6	14.0
≥ $85,000	16.6	13.3
**Risk factors, %**		
Abdominal obesity‡	87.2	90.4
Body mass index ≥ 28 kg/m^2^	74.7	75.9
BMI category, %*		
Underweight/normalweight (BMI ≤ 24.9 kg/m^2^)	14.1*	12.2
Overweight(BMI: 25.0–29.9 kg/m^2^)	29.9*	25.3
Obese (BMI ≥ 30 kg/m^2^)	55.9*	62.5
Hypertension diagnosis, %	84.6	85.0
Dyslipidaemia diagnosis, %	82.4	83.7
**Smoking, %**		
Current smoker	11.8	11.7
Past smoker	14.1	12.1
Never smoked	74.1	76.2
Physical activity§, %**		
Highly active	14.9**	9.3
Minimally active	20.9**	22.3
Inactive	64.2**	68.4

* p < 0.05;

** p < 0.001.

† 3% of each group had missing values for race.

‡ Waist circumference ≥ 97 cm for men and ≥ 89 cm for women.

§ International physical activity questionnaire score. HD, heart disease; SHIELD, Study to Help Improve Early evaluation and management of risk factors Leading to Diabetes; BMI, body mass index.

### CVD risk factors

Dyslipidaemia, hypertension and obesity were frequently reported by HD respondents with and without diabetes mellitus (Table 1). More than 55% of HD respondents without diabetes mellitus and 62% of HD respondents with T2DM were considered obese (defined as BMI ≥ 30 kg/m^2^), while > 87% of both groups had abdominal obesity. Significantly more HD respondents with T2DM were obese (BMI ≥ 30 kg/m^2^) than HD respondents without diabetes mellitus (p = 0.04) (Table 1). Approximately 12% of HD respondents with and without T2DM were current smokers. A large percentage (> 64%) of HD respondents were physically inactive as estimated by the IPAQ, and significantly more HD respondents with T2DM were inactive and fewer were highly active compared with the HD respondents without diabetes mellitus (p = 0.002).

### Age at HD diagnosis and time since diagnosis

Mean self-reported age at HD diagnosis among SHIELD respondents in the non-diabetes mellitus group was 56.8 years compared with 55.8 years in the T2DM group (p = 0.16) (Table 2). Respondents reported that they had HD for an average of 11.7 years in the non-diabetes mellitus group and an average of 10.7 years in the T2DM group (p = 0.06). Approximately 31% of HD respondents without diabetes mellitus and 36% of HD respondents with T2DM were diagnosed in 2001 or later (Table 2), during the period when a number of consensus statements and guidelines for screening and prevention of CHD and CVD were published (5,6,9,10,15,20). Between 1992 and 2000, a period during which several large studies provided evidence for primary and secondary prevention of CHD, including screening for risk factors (3,7,11), 37% of HD respondents without diabetes mellitus and 39% of HD respondents with T2DM were diagnosed. Before 1992, 31.9% of HD respondents without diabetes mellitus and 25.1% with T2DM were diagnosed. There were significantly more HD respondents without diabetes mellitus diagnosed earlier (1980s or earlier) than HD respondents with T2DM (p = 0.01).

**Table 2:** Age at diagnosis and year of diagnosis of HD in SHIELD respondents

Age and year of heart disease diagnosis	HD without diabetes (*n* = 973)	HD + type 2 diabetes mellitus (*n* = 600)
Age at heart disease diagnosis, mean (SD)	56.8 (13.5)	55.8 (13.7)
Years with heart disease, mean (SD)	11.7 (9.5)	10.7 (10.6)
Year of heart disease diagnosis, %*		
2004 or later	11.1*	17.3
2001–2003	19.9*	19.0
1998–2000	15.1*	14.7
1995–1997	13.4*	16.2
1992–1994	8.6*	7.7
1989–1991	9.4*	7.8
1986–1988	7.2*	5.3
1985 or earlier	15.3*	12.0

* p = 0.01 comparing HD respondents with and without type 2 diabetes mellitus. HD, heart disease; SHIELD, Study to Help Improve Early evaluation and management of risk factors Leading to Diabetes.

### Diagnosing HD

**Figure 2:**
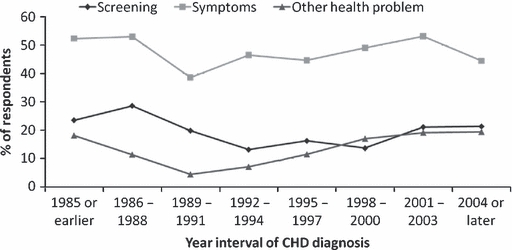
Method of HD diagnosis for SHIELD respondents with HD and no diabetes mellitus (*n* = 973). Respondents were permitted to check multiple responses

**Figure 3:**
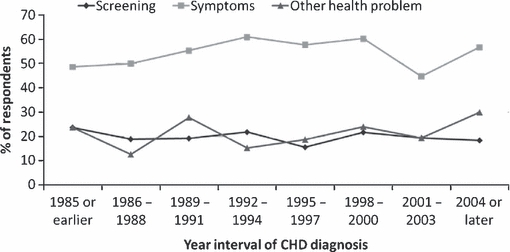
Method of HD diagnosis for SHIELD respondents with HD and type 2 diabetes mellitus (*n* = 600). Respondents were permitted to check multiple responses

In the non-diabetes mellitus group, 19.4% of individuals self-reported an HD diagnosis due to routine screening compared with 19.5% of the T2DM group (p = 0.99) (Table 3). However, a significantly greater proportion of HD respondents with T2DM reported the diagnosis based on having symptoms (54%) compared with respondents without diabetes mellitus (48%) (p = 0.03). In the non-diabetes group, 14.7% reported a diagnosis based on being treated for another health problem compared with 22.2% of the T2DM group (p = 0.0002) (Table 3). To determine if the method of diagnosis changed over time, the number of respondents reporting each method of diagnosis (screening, symptoms or other health problem) was stratified by the time interval in which their diagnosis of HD was made. The proportion of HD respondents without diabetes mellitus reporting a diagnosis based on symptoms fluctuated over time, yet the proportion reporting a diagnosis based on routine screening or other health problem had increased in recent years (1998 or later) but there was no significant trend over time (p > 0.05) (Figure 2). For respondents with HD and T2DM, the proportion reporting a diagnosis based on symptoms increased from 1985 to 1994 and then dropped, but increased again in recent years (2004 or later); however, the trend was not significant (p > 0.05) (Figure 3). The percentage of respondents with HD and T2DM reporting routine screening or other health problems as the method of diagnosis did not change over time (p > 0.05) (Figure 2).

**Table 3:** Method of diagnosis for HD and physician specialty diagnosing HD among SHIELD respondents

Method of diagnosis	HD without diabetes (*n* = 973)	HD + type 2 diabetes mellitus (*n* = 600)
During routine screening or blood test, %	19.4%	19.5%
Tested after having symptoms, %	48.3%*	54.0%
Tested during treatment for another health problem, %	14.7%*	22.2%
**Specialty of physician making diagnosis of HD**	*n* = 819	*n* = 569
Cardiologist, %	63.0%	68.7%
Family doctor/general practitioner, %	31.6%	27.1%
Endocrinologist, %	0.5%	0.9%
Other (neurologist, emergency room physician, pulmonologist, surgeon), %	4.9%	3.3%

* p < 0.05. HD, heart disease; SHIELD, Study to Help Improve Early evaluation and management of risk factors Leading to Diabetes.

### Specialty of physician who diagnosed HD

The majority of respondents with HD self-reported that they received their HD diagnosis from their cardiologist (63.0% and 68.7% in non-diabetes and T2DM groups, respectively) (Table 3). A smaller percentage indicated their family doctor or general practitioner as the physician diagnosing their HD (31.6% and 27.1% in non-diabetes mellitus and T2DM groups, respectively). Very few in either group (< 1%) reported that an endocrinologist had diagnosed their HD. Approximately 5% and 3% reported other physician specialties in the non-diabetes mellitus and T2DM groups, respectively. There was no significant difference between the groups in the specialty of physician making the HD diagnosis (p = 0.15). This pattern of physician specialty neither changed significantly over time nor did it differ substantially by respondent age group (p > 0.05).

.

## Discussion

This SHIELD analysis showed that the majority of respondents reported a diagnosis of HD based on symptoms; 48–54% of individuals with or without T2DM reported symptoms as the reason for the diagnosis of HD. Symptoms-based diagnosis was reported more frequently in the T2DM group, which may suggest that the opportunity to diagnose HD early (prior to symptoms) among this at-risk group is being missed for some individuals. In particular, the AHA/ADA scientific statement recommends risk factor screening annually (lipids) or at every routine diabetes visit (blood pressure) for those with T2DM (4). It also may indicate that respondents are not presenting for medical care until they have symptoms, resulting in a diagnosis of HD at an advanced stage of the disease. In either situation, increased educational efforts to promote awareness and screening of risk factors and detection of HD are warranted. However, there is some evidence that routine screening for HD is increasing in recent years among individuals without T2DM. The proportion of respondents with no diabetes mellitus reporting routine screening as the method of diagnosis increased from 1998–2000 to 2006. Also encouraging is the increase from 2001–2003 to 2006 for respondents with T2DM who reported their HD diagnosis was based on another health problem. This increase in the proportion diagnosed as a result of being treated for another health problem may be related to their diabetes mellitus care and is a good opportunity for identifying HD in this high-risk group. Yet, the increases in diagnosis through routine screening or other health problems were relatively small and not significantly different from the trend in prior years, so additional awareness-raising and adoption of the published guidelines are warranted, especially in light of the high prevalence of risk factors among these respondents.

Family practitioners often are the first healthcare providers an individual visits after experiencing symptoms of any kind, which may explain why up to one-third of respondents in this survey reported having a family practitioner diagnose them with HD. The majority of respondents, regardless of whether they had T2DM or not, were diagnosed with HD by a cardiologist. It is probable that these respondents were referred to the cardiologist by their primary care physician (or endocrinologist or emergency care physician) for further evaluation, where the diagnosis was ultimately made.

Findings from the SHIELD surveys also confirm that respondents with HD present with one or more of the key risk factors associated with CHD and CVD. For both HD respondents with and without T2DM, the majority had dyslipidaemia and hypertension and were overweight or obese. The T2DM group reported higher obesity rates, which was expected, as individuals with T2DM are more likely to be overweight or obese (21). Of note, < 12% of those with an HD diagnosis indicated that they were current smokers in either group, which may indicate increased awareness of smoking and its contribution to CHD and CVD. However, it is not known whether the individuals were smoking at the time of their HD diagnosis. Only 15% of the non-diabetes mellitus group and 9% of the T2DM group self-reported that they were exercising regularly (highly active), and there were more inactive individuals with a self-reported diagnosis of HD and T2DM than those with a self-reported diagnosis of HD without diabetes mellitus, which may indicate that individuals are not aware of the importance of exercise in reducing their HD risk (22).

National data indicate that the average age of patients experiencing a myocardial infarction is approximately 66 years for men and 70 years for women (1). In the SHIELD study, respondents with and without T2DM reported an average age at diagnosis of HD of 56–57 years, which might indicate that these respondents are being diagnosed earlier, possibly before their first myocardial infarction. In addition, significantly more respondents with T2DM were diagnosed in recent years (2001 or later) compared with respondents without diabetes mellitus, which may indicate greater awareness of the cardiovascular risk that diabetes mellitus poses, possibly through the publication and adoption of the guidelines from AHA, ACC and NCEP.

This study provides evidence of the methods employed for and the physician specialties diagnosing HD in a large sample of respondents with a high survey rate who are representative of the US population. However, there are limitations to the study that should be considered. Only a small percentage (5–8%) of those invited to participate in the TNS NFO panel elect to do so, and those who participate are accustomed to completing surveys, leading to the possibility of selection bias. Household panels tend to under-represent the very wealthy and very poor segments of the population and do not include military and institutionalised individuals, which are shortcomings for most random sampling and clinically based studies. Additionally, the determination of HD, diabetes mellitus and risk factors was made based upon self-report rather than clinical or laboratory measures for blood glucose, cholesterol and hypertension. Recall of method of diagnosis by the respondent also could potentially differ for recently diagnosed respondents compared with respondents given the diagnosis more than 15 years previously. There may be potential for recall bias; however, the trends for methods of diagnosis did not change significantly between 1992 and 2000, which may indicate similar recall among those diagnosed 15–16 years ago and those diagnosed 7–8 years ago. Recall bias may potentially affect those diagnosed more than 15 years ago.

## Conclusions

Despite increased knowledge and awareness of the risk factors for CHD, many individuals are not diagnosed with HD until they are symptomatic. The fact that only a small percentage of SHIELD respondents were diagnosed through screening indicates that there is a missed opportunity to diagnose HD during earlier, less severe stages of the disease. As blood pressure and weight are evaluated at most physician office visits, medical providers already have information on two key modifiable risk factors. There is a need for improved targeted education toward patients and physicians on reducing HD risk before symptoms occur.
